# Editorial: Causes, effects and treatment of violence and aggression in mental health and social care settings

**DOI:** 10.3389/fpsyt.2022.935821

**Published:** 2022-08-09

**Authors:** Brian Littlechild

**Affiliations:** University of Hertfordshire, Hatfield, United Kingdom

**Keywords:** mental health, aggression, violence, causes, effects, treatment

Interpersonal violence is widespread within societies and has historically often been a neglected issue in policy, law and practice. Avoidance of acknowledging and dealing with the causes and effects of such interpersonal violence in many different settings in societies across the globe has far too often been the case. This includes within family relationships where abuse and violence, with such violence frequently still taking place. It also includes institutional settings, where there also is current evidence of violence. A number of countries and agencies around the world are now more seriously starting to confront the difficult task of educating individuals and agencies within their societies, with lawmakers and policy makers now in some areas starting to deal with and attempting to reduce this problem. It can be argued that this is partly due to the bravery of survivors of such violence and professionals in the field coming forward to air their experiences and concerns, and their voices being heard through the media, politicians, and researchers, which has highlighted evidence from survivors, campaigners and researchers giving attention to the enormous scale and severity of the problem in mental health, learning disability, and children and young people's institutions, for example. In recent years, there has been more attention paid to this area, not least of all within mental health settings and provision, particularly in inpatient mental health settings, although there is less evidence about this in relation to community settings. The scope and meaning of violence and aggression in this arena covers abuse, aggression and violence in interpersonal relationships. We know that the effects of such behaviors in mental health settings can negatively affect the health, wellbeing, and feelings of security and safety of both service users and staff, making this an important area to explore further, examining ways to not only reduce the number of such incidents, by way of greater understanding of the causes of such violence and aggression, from the basis of pharmacological, psychosocial and organizational causes of and responses to such behaviors.

From such a set of understandings, this Special Issue also considers how best to deal with these situations, and the effects on service users and staff.

The aim of this Research Topic is to address these matters in a systematic way, in order to provide evidence and lessons in advancing knowledge and skills so as to prevent and treat the often very serious effects on people mental health problems that relate to such situations. In so doing, the collection of articles covers a range of areas and issues, including the areas such as the ways in which childhood abuse and neglect can increase the risk of both mental disorders and violent behavior, and the ways in which frontline healthcare staff have been affected by workplace violence during the pandemic, and consequent effects on their wellbeing and levels of anxiety. Ways in which drug use might affect violent behavior is also examined within one of the articles, whilst the effects on current aggressive and violent behavior of diagnosed mental health problems and previous traumas are highlighted in two further articles. A focus on how managers in mental health settings best deal with the issues of violence and aggression is dealt with in two of the articles. In one of these, the focus is on how a strengthened emphasis may be taken, based on a value-based approach to such violence is seen and responded to, whilst a further one demonstrates from one particular project that found that de-escalation training can be effective in reducing the incidence and severity of aggression in acute psychiatric units, and the use of physical restraints.

In reviewing the content and lessons to be learnt from these articles, further work in this area could include particular issues relating to certain psychiatric diagnoses such as schizophrenia, impulse control disorder, ADHD, conduct disorder and antisocial personality disorder, and precipitating and protective factors; institutional policies in different countries, and human rights issues.

We put forward this collection of articles with the intention and hope that it will provide ideas and findings for those in academic, policy, management and practice areas to have access to an enhanced understanding of the causes, effects, and best means of trying to reduce both the incidents and effects of such violence and aggression, to the benefit of both patients and staff alike.

## Author contributions

The author confirms being the sole contributor of this work and has approved it for publication.

## Conflict of interest

The author declares that the research was conducted in the absence of any commercial or financial relationships that could be construed as a potential conflict of interest.

## Publisher's note

All claims expressed in this article are solely those of the authors and do not necessarily represent those of their affiliated organizations, or those of the publisher, the editors and the reviewers. Any product that may be evaluated in this article, or claim that may be made by its manufacturer, is not guaranteed or endorsed by the publisher.

